# Blocking COX-2 induces apoptosis and inhibits cell proliferation via the Akt/survivin- and Akt/ID3 pathway in low-grade-glioma

**DOI:** 10.1007/s11060-017-2380-5

**Published:** 2017-03-10

**Authors:** Aya Sato, Yoshifumi Mizobuchi, Kohei Nakajima, Kenji Shono, Toshitaka Fujihara, Teruyoshi Kageji, Keiko Kitazato, Kazuhito Matsuzaki, Hideo Mure, Kazuyuki Kuwayama, Akiko Sumi, Hideyuki Saya, Oltea Sampetrean, Shinji Nagahirao

**Affiliations:** 1 Department of Neurosurgery, Institute of Biomedical Sciences, Tokushima University Graduate School, 3-18-15, Kuramoto-cho, Tokushima, 770-8503 Japan; 2 Division of Gene Regulation, Institute for Advanced Medical Research, Keio University School of Medicine, Tokyo, Japan

**Keywords:** COX-2, Low-grade glioma, Akt, Survivin, ID3

## Abstract

Approximately half of surgically-treated patients with low-grade-glioma (LGG) suffer recurrence or metastasis. Currently there is no effective drug treatment. While the selective COX-2 inhibitor celecoxib showed anti-neoplastic activity against several malignant tumors, its effects against LGG remain to be elucidated. Ours is the first report that the expression level of COX-2 in brain tissue samples from patients with LGG and in LGG cell lines is higher than in the non-neoplastic region and in normal brain cells. We found that celecoxib attenuated LGG cell proliferation in a dose-dependent manner. It inhibited the generation of prostaglandin E2 and induced apoptosis and cell-cycle arrest. We also show that celecoxib hampered the activation of the Akt/survivin- and the Akt/ID3 pathway in LGGs. These findings suggest that celecoxib may have a promising therapeutic potential and that the early treatment of LGG patients with the drug may be beneficial.

**Electronic supplementary material:**

The online version of this article (doi:10.1007/s11060-017-2380-5) contains supplementary material, which is available to authorized users.

## Introduction

Low-grade gliomas [LGGs, World Health Organization (WHO) grade I or II] are a diverse group of primary brain tumors that often arise in young, otherwise healthy individuals. Their course is indolent and the survival of patients with LGG tends to be longer than of patients with high-grade gliomas [WHO grade III or IV, glioblastoma multiforme (GBM)]. Treatment options include observation, surgery, radiation, chemotherapy, and a combined approach. Patient management is individualized and depends on the tumor location, histology, molecular profile, and the patient characteristics [1].

Compared to WHO grade III anaplastic astrocytomas (AAs) and GBMs, LGGs including diffuse astrocytoma, oligodendroglioma, and oligoastrocytoma have a highly variable prognosis. Although some patients survive for decades, LGGs progress in an infiltrative manner and transform into malignant tumors such as AA or secondary GBM [1, 2]. Therefore, effective treatment strategies must be developed. The possible pharmaceutical management of LGGs and the importance of prognostic factors for survival remain uncertain.

Nonsteroidal anti-inflammatory drugs (NSAIDs) have been widely used to treat various diseases. Traditional NSAIDs including aspirin, indomethacin, diclofenac, and sulindac inhibit both COX-1 and COX-2 activity; new-generation drugs such as celecoxib and rofecoxib selectively inhibit COX-2 activity [3, 4]. In GBM cell lines, celecoxib induced apoptosis and inhibited cell proliferation and angiogenesis [4]. However, there are few studies on the anti-tumor effects of celecoxib in LGG.

Survivin belongs to the family of proteins that inhibits apoptosis; in various cancer cells it inhibited apoptosis and induced their proliferation [5]. These effects of survivin are associated with Akt and prostaglandin E2 (PGE2) [6].

The expression of COX-2 was also associated with inhibitor-of-differentiation (ID) proteins that are significantly increased in several cancer cell lines [7]. ID1 to ID4 are in a group of basic helix-loop-helix (bHLH) proteins that lack a DNA-binding domain. They inhibit the DNA-binding activity of bHLH transcription factors via heterodimerization [8]. Although they are cell-fate determinants and involved in a broad range of processes associated with tumorigenesis, the ID subtype in LGGs affected by celecoxib remains to be identified. Under the hypothesis that celecoxib exerts anti-tumor effects in LGG cells, we investigated the mechanisms underlying these effects.

Here we show that the expression level of COX-2 in LGG cells is higher than in the non-neoplastic region (NNR) of the brain and in normal human astrocytes (NHA). We demonstrate that celecoxib-treatment of LGG cells induces apoptosis and inhibits their proliferation via the Akt/survivin- and the Akt/ID3 pathway.

## Materials and methods

### Tissue samples

Our study was approved by the Ethics Committee of Tokushima University Graduate School; patient informed consent for the use of tissue samples obtained at surgery in the Department of Neurosurgery was also acquired. We studied 12 tissue samples, five were from glioblastoma- and seven from glioma patients. All samples were classified by neuropathologists according to the WHO classification of brain tumors. The sections and lysates from NNR were purchased from BioChain Institute (Newark, NJ, USA). Each tissue sample was subjected to immunohistochemical assessment and Western blot analysis.

### Cell lines

Human glioma- and GBM cell lines (1321N1, SW1088, U87MG, U251MG, and NHA) were purchased from the European Collection of Cell Cultures (Salisbury, Wiltshire, UK), American Type Culture Collection (Manassas, VA, USA), the Health Science Research Resources Bank (Osaka, Japan), and Lonza Japan Co. (Tokyo, Japan). The 1321N1, SW1088 and GBM cell lines were cultured in RPMI-1640 medium (Invitrogen, NJ, USA) or in DMEM high-glucose medium (Wako, Osaka, Japan) with 10% fetal bovine serum (GIBCO-BRL, NY, USA). NHA were cultured using the AGM™ BulletKit™ according to the manufacturer’s protocol.

### Cell viability assay

Plated cells were treated with 1% DMSO (vehicle control, VC) or with 10-, 20-, or 100 µM celecoxib once (on day 1), twice (on days 1 and 2), or three times (on days 1, 2 and 3). The controls were not exposed to celecoxib. Viable cells were assessed using WST-8 reagent (Dojindo, Kumamoto, Japan) and a microplate reader (TECAN, InfiniteR 200 PRO, Kanagawa, Japan); cell viability (%) was calculated as the relative ratio to VC.

### Malignant mouse brain tumor model

Genetically modified GFP-labeled glioma stem cells were established by Saya et al. [9] at Keio University and kindly provided to induce malignant brain tumors. Cultured cells were orthotopically transplanted into 6–8-week old anesthetized male C57BL/6 mice according to Sampetrean et al. [10]. Using a 10-ml Hamilton syringe, 1000 viable cells in 2 ml of Hank’s balanced salt solution were stereotactically injected into the right hemisphere, 3 mm below the brain surface, of anesthetized mice. They were monitored daily for the development of neurological deficits and deaths on days 24–35 were recorded. Based on our preliminary dose-determination study, we used 10 mg/kg celecoxib and assessed its effects on the tumor size.

### Immunohistochemical staining

Immunohistochemical staining with the VECTASTAIN ABC kit (Vector Laboratories, San Mateo, USA) was performed according to the manufacturer’s protocol. After antigen retrieval with EDTA (pH 9.0), inactivation in 3% H_2_O_2_/MeOH, and blocking with protein-block (Dako Japan), the antigen was incubated with rabbit polyclonal anti-COX-2 antibody (abcam, Cambridge, UK) at a 1:2000 dilution with Can Get Signal Solution 1 (Toyobo, Osaka, Japan). This was followed by incubation with the biotinylated secondary antibody and the VECTASTAIN ABC reagent.

### Assessment of cell-cycle arrest

After fixing with 4% paraformaldehyde, LGG cells were incubated with rabbit polyclonal anti-Ki67 antibody (abcam) at a 1:1000 dilution in PBS in the presence of 1% bovine serum albumin. The antigen was detected with Alexa FlourR488 goat anti-rabbit IgG antibody (Invitrogen, Carlsbad, CA, USA). We used 4′,6-diamino-2-phenylindole (DAPI) (Dojindo, Kumamoto, Japan) and observed the cells under a fluorescence microscope (KEYENCE, BZ710, Osaka, Japan).

### Annexin V assay

Annexin V-positive cells were detected with the Annexin V assay kit (BioVision, CA, USA) according to the manufacturer (NCE, BZ710, Osaka, Japan). The 0.1% DMSO or 20 µM celecoxib on days 1 and 2 were, or were not, exposed for 12 h to 20 µM of the pan-caspase inhibitor boc-aspartyl-(OMe)-fluoromethyl-ketone (BAF) (MP Biochemicals, OH, USA) and stained with annexin V-FITC and DAPI.

### Western blot analysis

The protein concentration in cell lysates was assayed with the BCA reagent (Thermo Scientific, IL, USA). Protein (15–50 µg) was separated by SDS–PAGE and transferred to polyvinylidene fluoride (PVDF, BIO-RAD, CA, USA) membranes. After blocking, the membranes were incubated with the primary antibodies (Fig. S1) in CanGet Signal Solution 1and then with horseradish peroxidase-conjugated secondary antibodies in Can Get Signal Solution 2. The protein-antibody complexes were detected with Amersham ECLR or ECLR plus (GE Healthcare, UK) using a Lumino image analyzer (Image Quant LAS4000 mini, GE Healthcare, UK) and NIH ImageJ 1.46 software. Each experiment was repeated three times.

### Prostaglandin E2 (PGE2) ELISA assay

The culture medium was collected, concentrated using Microcon^R^ultracel-30 membranes (Millipore, MA, USA), and the PGE2 level was determined with the PGE2 high-sensitivity ELISA kit (ENZO Life Sciences, NY, USA) according to the manufacturer’s protocol.

### Statistical analysis

All data (mean ± SD) were analyzed with the Student *t* test for two-group comparisons. Statistical analyses were performed using Excel 2010 (Microsoft, Seattle, WA) with add-in software (Statcel 3; OMS, Saitama, Japan). Differences of p < 0.05 were considered statistically significant.

## Results

### COX-2 expression is increased in brain regions affected by LGG and in LGG cells

**Fig. 1:**
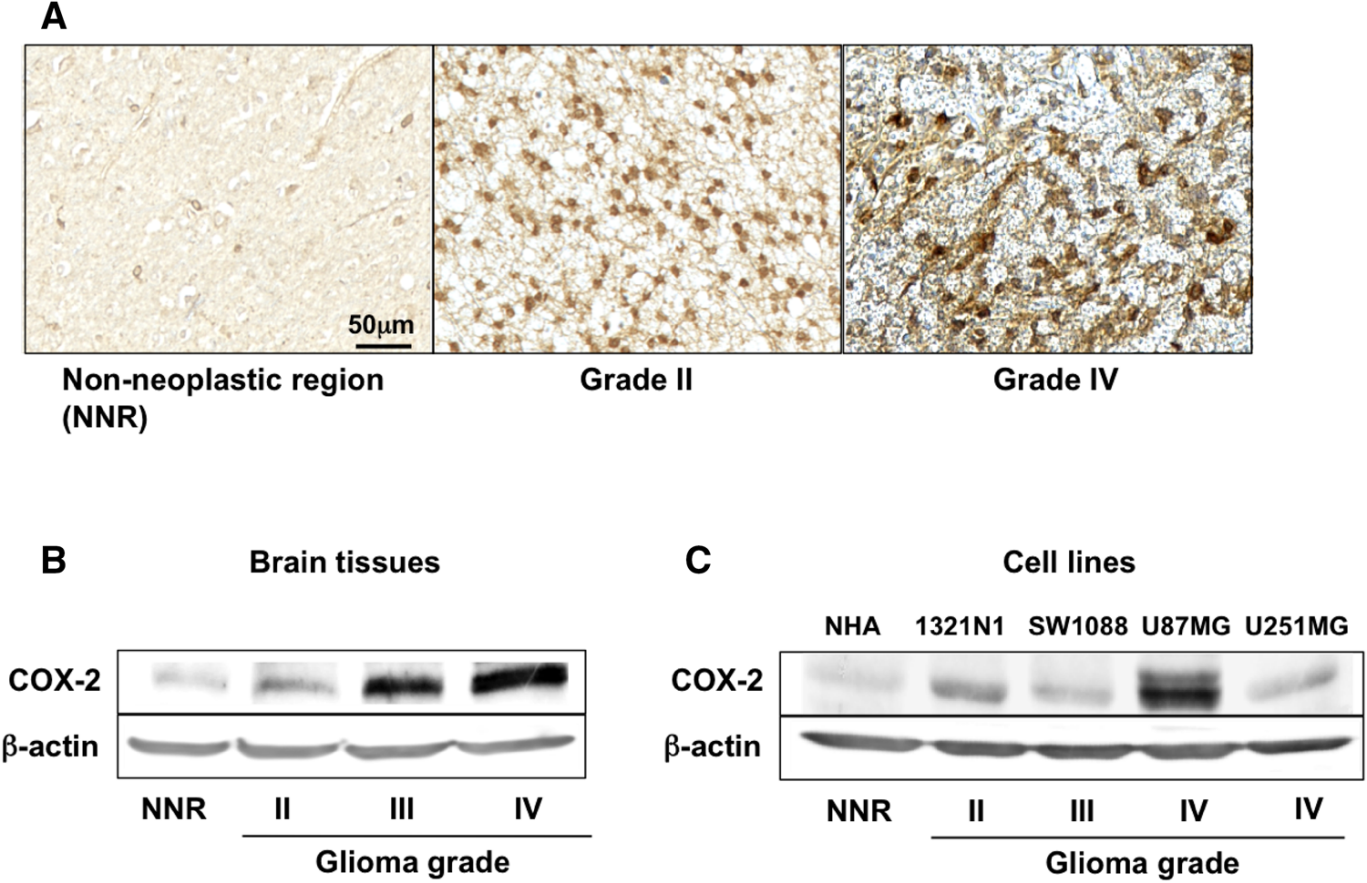
COX-2 protein expression in glioma tissue and cells. **a** Representative immunohistochemical staining for COX-2 protein in normal brain- (n = 2) and glioma samples (n = 12). *Scale bar* 50 µm. **b** The COX-2 protein level determined by Western blot analysis was analyzed in control-(non-neoplastic region, n = 2), astrocytoma-(glioma grade II, n = 2), anaplastic astrocytoma-(glioma grade III, n = 5), and glioblastoma (glioma grade IV, n = 5) samples. **c** COX-2 expression determined by western blot analysis was analyzed in glioma cell lines (1321N1, SW1088, U251MG, U87MG) and normal human astrocytes (NHA). Each experiment was repeated three times

Immunohistochemically, the expression of COX-2 was higher in brain regions with WHO grade II glioma than in NNR; the higher the glioma grade, the higher was COX-2 expression (Fig. 1a). Western blot analysis yielded the same results (Fig. 1b). The expression of COX-2 protein in glioma cell lines (1321N1, SW1088, U87MG, U251MG) was higher than in NHA; its expression also increased with the glioma grade (Fig. 1c).

### Celecoxib reduces PGE2 generation and the viability of LGG cells

**Fig. 2:**
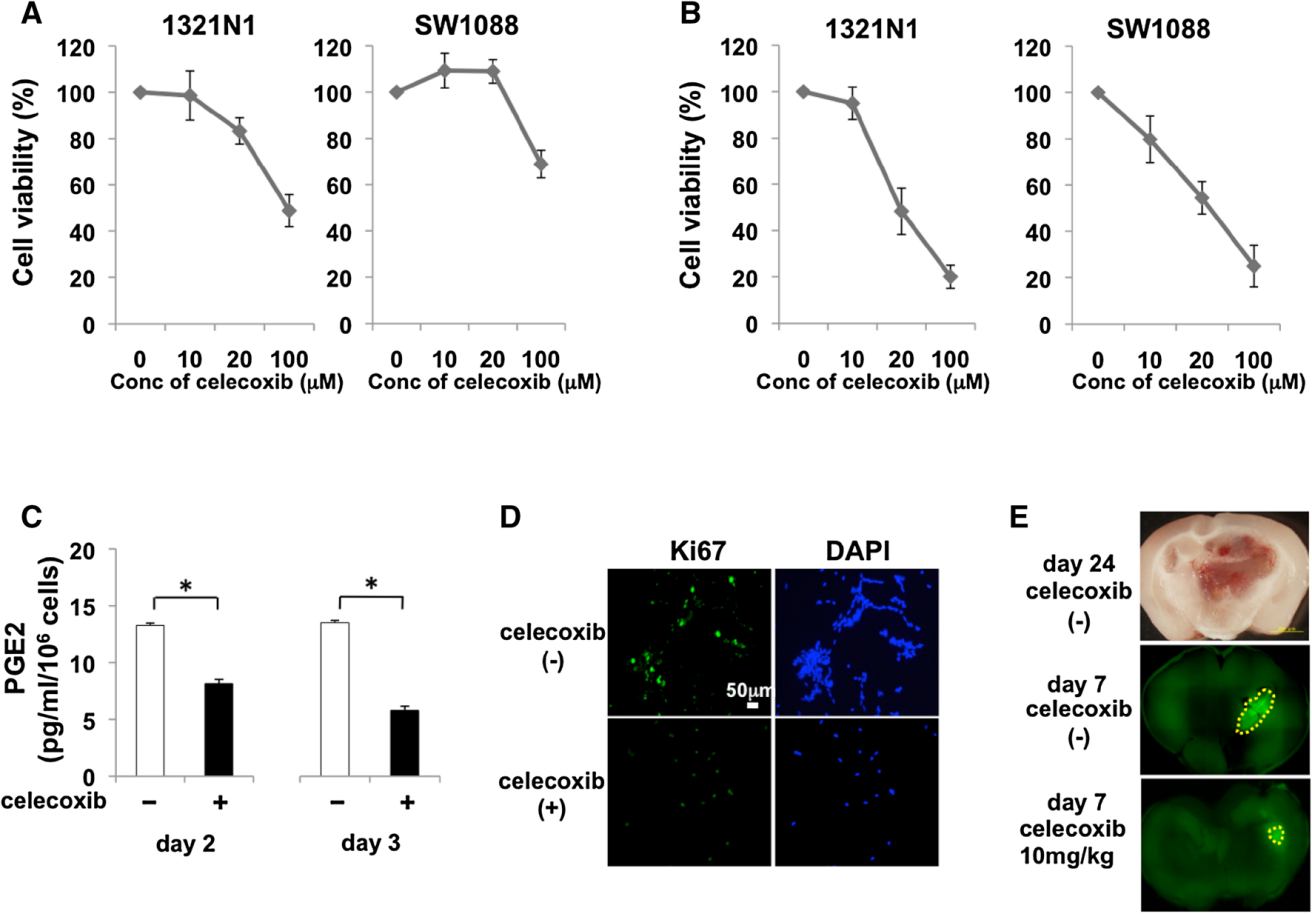
The effect of celecoxib on cell viability and the PGE2 level in LGGs. **a** Viability of cells exposed, or not exposed, to single treatment with celecoxib. Cell viability was determined by MTT assay 24 h post-treatment with the indicated concentrations of celecoxib dissolved in 0.1% DMSO. **b** Viability of cells exposed, or not exposed, to triple treatment with celecoxib. **c** Using ELISA, PGE2 expression was measured in 1321N1 cells that were, or were not, exposed 2 (days 1 and 2) or 3 times (days 1, 2, 3) to 20 µM celecoxib. Data are the mean ± SD (n = 6). Celecoxib-treated vs -untreated cells, *p < 0.05 by Student’s *t* test. **d** The expression of Ki67 in 1321N1 cells untreated or treated three times with 20 µM celecoxib was detected by immunostaining. The cell nuclei were counterstained with DAPI (*blue*). Each experiment was repeated three times. **e** *Top* representative malignant tumor harvested 24 days after the orthotopic transplantation of genetically modified stem cells and GFP-labeled stem cells. After transplantation, the murine host was (*bottom*), or was not (*center*), treated for 7 days with 10 mg/kg/day celecoxib and the tumor sizes were assessed on day 7

The viability of 1321N1- and SW1088 cells was inhibited by one- (Fig. 2a) and by 3-day treatment (Fig. 2b) with celecoxib in a dose-dependent manner. The generation of PGE2 was inhibited by celecoxib on day 2 and day 3 (Fig. 2c). Immunohistochemically, Ki-67 expression was attenuated by celecoxib in 1321N1 cells (Fig. 2d) and the expression of cyclin D1 was reduced (data not shown). These observations suggest that the celecoxib-induced inhibition of PGE2 generation elicited apoptosis and attenuated LGG cell proliferation. To date, no LGG animal model has been established. We used glioma model established by Saya et al. [9, 10] to examine the anti-tumor effect by celecoxib. On day 24, the model mice harbored malignant glioma (Fig. 2e). When celecoxib was added in the early phase after orthotopic glioma cell transplantation in the glioma model mice, the tumor size was smaller in mice treated for 7 days with 10 mg celecoxib/kg than in untreated mice (Fig. 2e).

### Celecoxib induces apoptosis in LGG cells

**Fig. 3:**
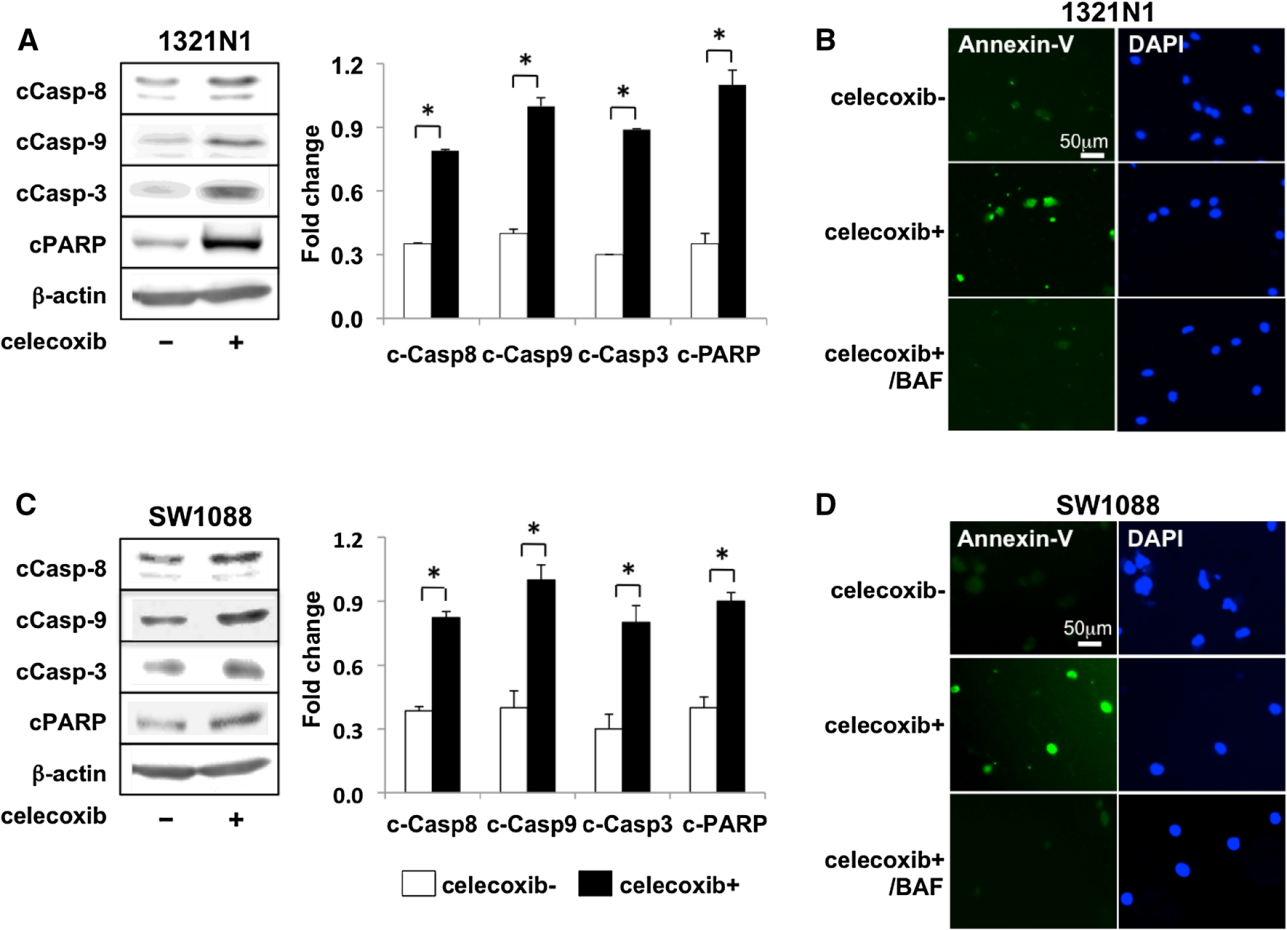
Caspase-dependent apoptosis induced by celecoxib in LGGs. **a** The expression of cleaved caspase-8 (cCas-8), -9, -3, and poly-ADP ribose polymerase (cPARP) was examined 3 h after the second treatment of 1321N1 cells with 20 µM celecoxib. **b** 1321N1 cells treated 6 h earlier with 20 µM celecoxib with or without BAF were stained with Annexin V-FITC and DAPI. **c**, **d** Western blot analysis and Annexin V staining of SW1088 cells. Each experiment was repeated three times. Each image was analyzed using LAS4000 and NIH Image J software. The results were normalized to β-actin. Data are the mean ± SD (n = 3). Celecoxib-treated vs -untreated cells, *p < 0.05 by Student’s *t* test

We further examined the effect of celecoxib on the apoptosis pathway. The expression of cleaved caspase, cCas-8, -9, -3, and of cleaved PARP (cPARP) was increased in 1321N1 and SW1088 cells (Fig. 3a, c). Annexin V assay with or without the caspase-specific inhibitor BAF showed that celecoxib treatment increased the number of Annexin V-positive 1321N1 and SW1088 cells; this increase was abrogated in the presence of BAF (Fig. 3b, d). These observations indicate that treatment with celecoxib led to intrinsic- and extrinsic pathway-dependent apoptosis in both cell lines.

### Celecoxib inhibits the activation of Akt and survivin expression in LGG cells

**Fig. 4:**
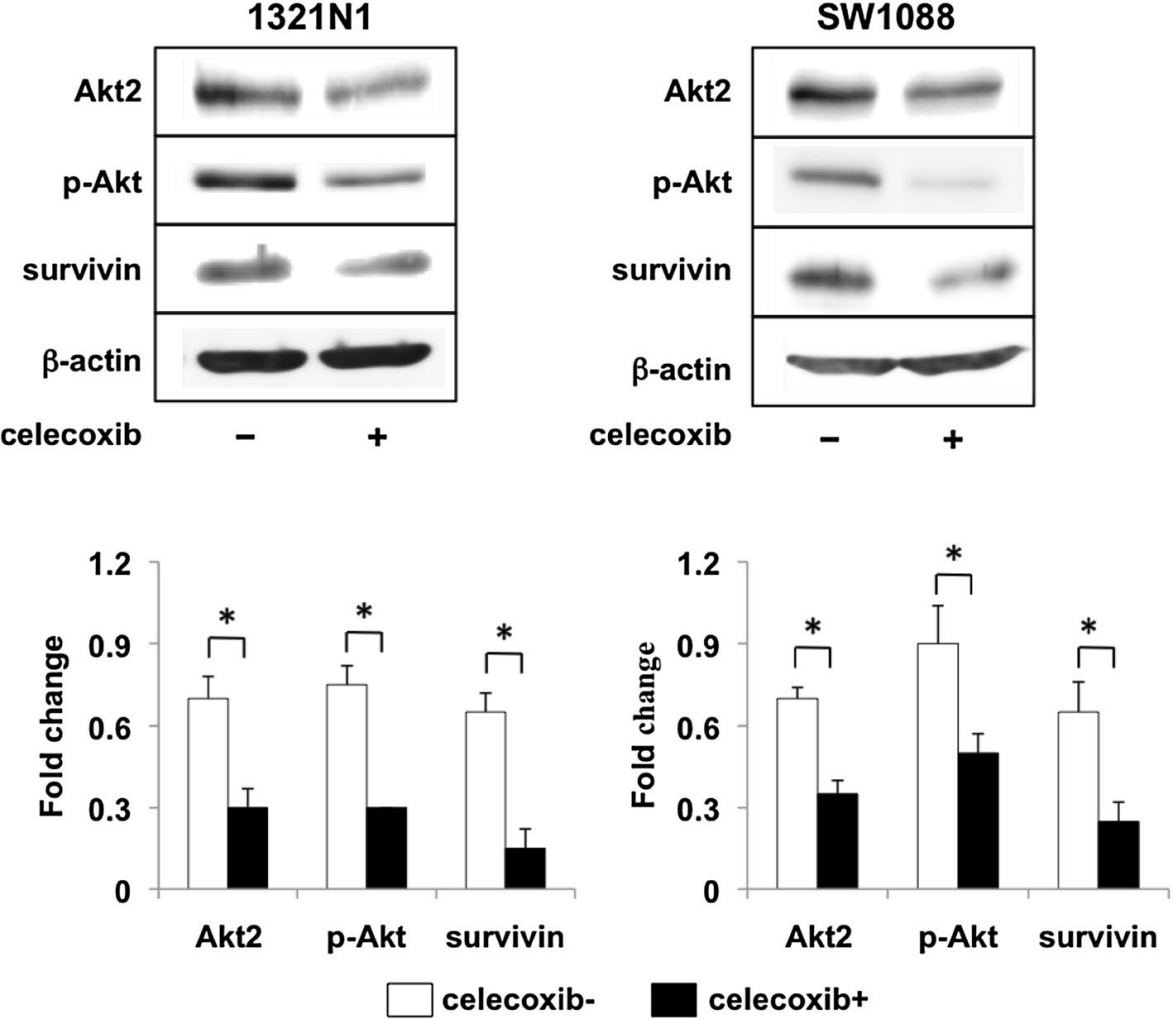
Expression of cell Akt/survivin pathway-related proteins in LGG cells treated, or not treated, with celecoxib. Representative western blot showing the expression of AKT2, p-AKT, and survivin in 1321N1 and SW1088 cells after triple treatment with 20 µM celecoxib. The expression of c-Myc, Bcl-2, cyclinD1 is also shown. Data are the mean ± SD (n = 3). Celecoxib-treated vs -untreated cells, *p < 0.05 by Student’s *t* test

The expression of survivin was up-regulated by Akt activation in glioma cells; celecoxib inhibited the activation of Akt [11, 12]. The expression of Akt2 protein, Akt2 and Akt phosphorylation, survivin expression were lower in both cell lines treated with celecoxib than celecoxib-untreated controls (Fig. 4).

### Inhibition of Akt activation by celecoxib leads to the reduction of survivin and ID3 protein

**Fig. 5:**
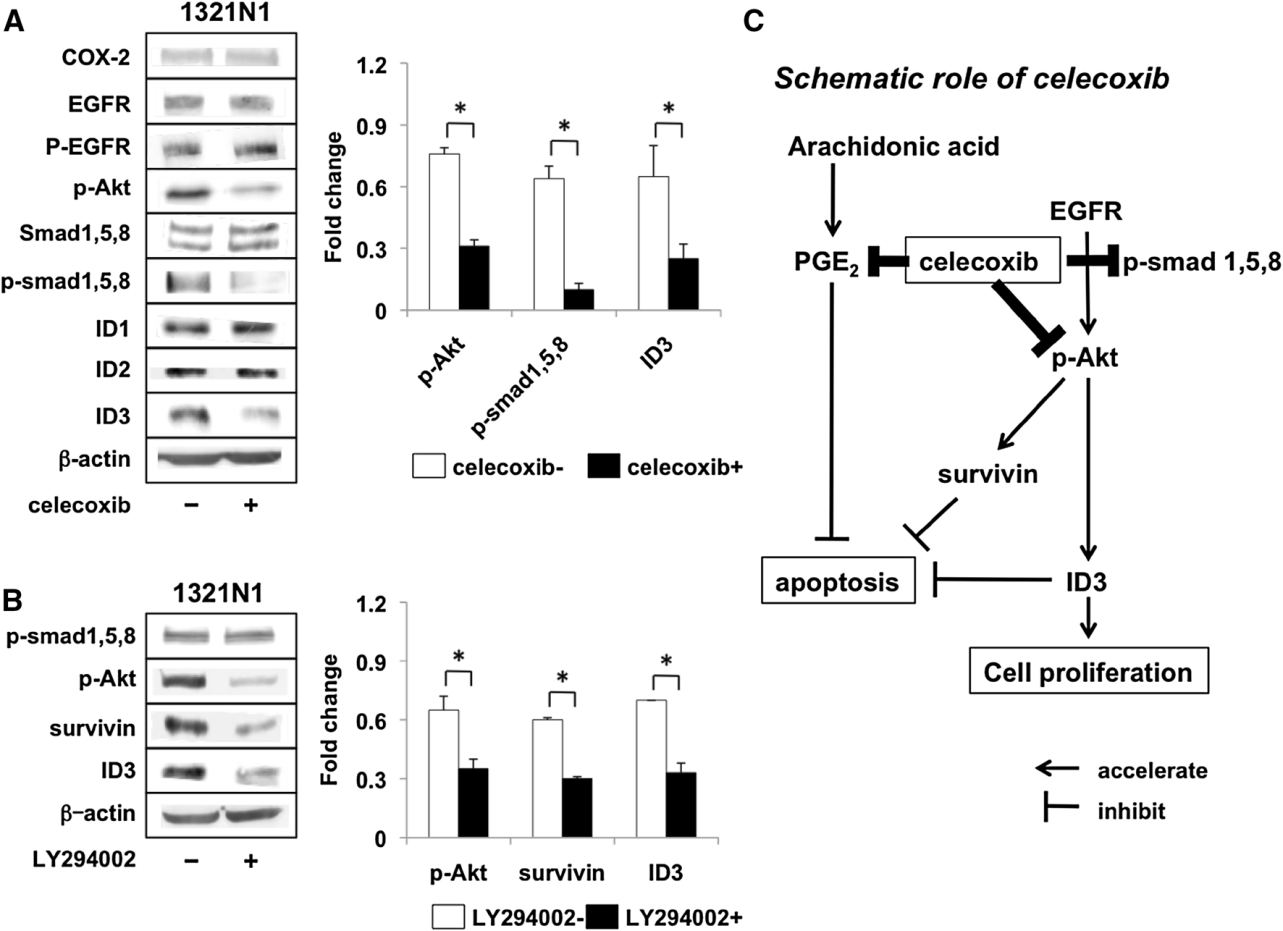
Inhibition of Akt activation and expression of ID3 protein elicited by celecoxib in 1321N1 cells. **a** Akt, smad1, -5, -8 phosphorylation and the expression of ID 1–3 after triple treatment with 20 µM celecoxib. **b** Phosphorylation of Akt, smad1, -5, -8 and the expression of ID3 in 1321N1 cells treated for 1 h with 50 µM of a PI3K/Akt inhibitor (LY294002). Each experiment was repeated three times. Data are the mean ± SD (n = 3). Celecoxib-treated vs -untreated cells, *p < 0.05 by Student’s *t* test

Jin et al. [13] reported that EGFR-Akt-Smad signaling promoted the formation of glioma stem-like cells and tumor angiogenesis mediated by ID3-derived cytokine induction. The expression of the ID family was related to the expression of COX-2 [7]. We found that the phosphorylation of Smad-1, -5, -8, and ID3 protein was attenuated by celecoxib (Fig. 5a). No other ID- and COX-2 proteins, nor the expression of EGFR were changed by the exposure of LGG cells to celecoxib. To further investigate whether the effects of celecoxib on Smad-1, -5, -8, and ID3 are mediated by Akt, we used the Akt/PI3K inhibitor LY294002 (Fig. 5b). Akt phosphorylation and the expression of survivin and ID3 were decreased by LY294002 without affecting Smad-1, -5, and -8 phosphorylation. Our observations suggest that celecoxib inhibited the Akt/survivin and the Akt/ID3 pathway (Fig. 5c).

## Discussion

We found that the expression of COX-2 is higher in LGG tissue and LGG cells than in the brain NNR and in NHA and that the PGE2 increase in LGG cells was attenuated by celecoxib. We demonstrated that celecoxib decreased the expression of Ki67 in LGG cells and that treatment with celecoxib in the early phase after orthotopic tumor cell transplantation decreased the tumor size in mice. We also showed that celecoxib induced caspase-dependent apoptosis and inhibition of the cell survival pathway mediated by the Akt/survivin pathway in LGG cells. In addition, we report that celecoxib inhibited the Akt/ID3 pathway. Our findings suggest that inhibition of PGE2 generation and of the Akt/survivin- and the Akt/ID3 pathway by celecoxib may help to prevent LGG tumorigenesis.

The expression of COX-2 in human glioma and its in vitro inhibition by a COX-2 inhibitor was reported by Joki et al. [14]. As various tumors secrete high levels of proinflammatory cytokines and PGE2, a local proinflammatory microenvironment can be elicited. Chronic inflammation contributes to tumor onset and progression [15]. On the other hand, selective COX-2 inhibition leads to a reduction in PGE2 synthesis and results in a marked anti-neoplastic effect on hepatocellular carcinoma cells; it has been associated with a significant induction of apoptosis [16, 17]. Consistent with such findings, in LGG cells we observed a reduction in PGE2 by celecoxib and the induction of apoptosis. The inhibition of PGE2 may hamper the viability of LGG cells.

Apoptosis elicited by celecoxib can be induced by the extrinsic- or the intrinsic pathway. Both require the activation of caspases that cleave various proteins and activate DNAses, resulting in DNA fragmentation [18, 19]. The apoptosis pathway may be activated by celecoxib in different cell types and cancers. As the drug increased the expression of cCas-8, -9, -3, and cPARP in 1321N1 and SW1088 cells, it appears to induce both extrinsic- and intrinsic pathway-dependent apoptosis. Elucidation of the detailed mechanisms underlying apoptosis induction requires further study.

The inhibition of PKB by celecoxib reduces all of its pathway activities and promotes apoptosis [19]. Our and studies by others [20, 21] have shown that the inhibition of Akt2 and Akt3 activates the intrinsic apoptotic pathway mediated by mitochondria in GBM cell lines [20, 21]. Dandekar et al. [22] reported that celecoxib-induced apoptosis in prostate cancer cells appeared to be accompanied by a reduction in the expression of anti-apoptotic proteins Bcl-2, Bcl-xL, and survivin. While the expression of survivin is increased in glioma cells [23], its level was reduced via the inhibition of Akt activation in glioblastoma cells [11]. We document that celecoxib inhibited the Akt/survivin pathway in LGG.

ID proteins are transcriptional regulators that control the timing of cell-fate determination and differentiation in stem- and progenitor cells during normal embryonal development and in adult life. ID proteins are associated with carcinogenesis and COX-2 [7]. ID3 knockdown decreased the proliferation and increased apoptosis of medulloblastoma cells [24] and ID3 was associated with the EGFR-Akt pathway [13, 25]. In our study celecoxib inhibited the expression of ID3 through Akt phosphorylation but it did not inhibit EGFR and the other ID family proteins, suggesting the EGFR-independent regulation of Akt downstream by celecoxib in LGG cells (Fig. 5c). Although we cannot rule out other mechanisms underlying the anti-tumor effects of celecoxib, the reduction in PGE2 and the inhibition of Akt pathway activation may represent a promising strategy for treating LGGs. To ascertain its clinical applicability we need further studies to confirm the efficacy of celecoxib in animal models of LGG tumors.

In conclusion, we first demonstrate that treatment with celecoxib exerts anti-tumor effects in cultured LGG cells. We also show that the inhibition of Akt activation by celecoxib led to the reduction of survivin and ID3 protein. Our findings suggest that LGGs may be treatable by drugs and may lay the groundwork for the future standardized treatment of these tumors.

## Electronic supplementary material

Below is the link to the electronic supplementary material.

Supplementary material 1 (DOCX 225 KB)
