# Supplementary figures and images for: Blocking COX-2 induces apoptosis and inhibits cell proliferation via the Akt/survivin- and Akt/ID3 pathway in low-grade-glioma
Source: J Neurooncol. 2017 Mar 10;132(2):231–8. doi: 10.1007/s11060-017-2380-5 (PMC6763415; doi:10.1007/s11060-017-2380-5)

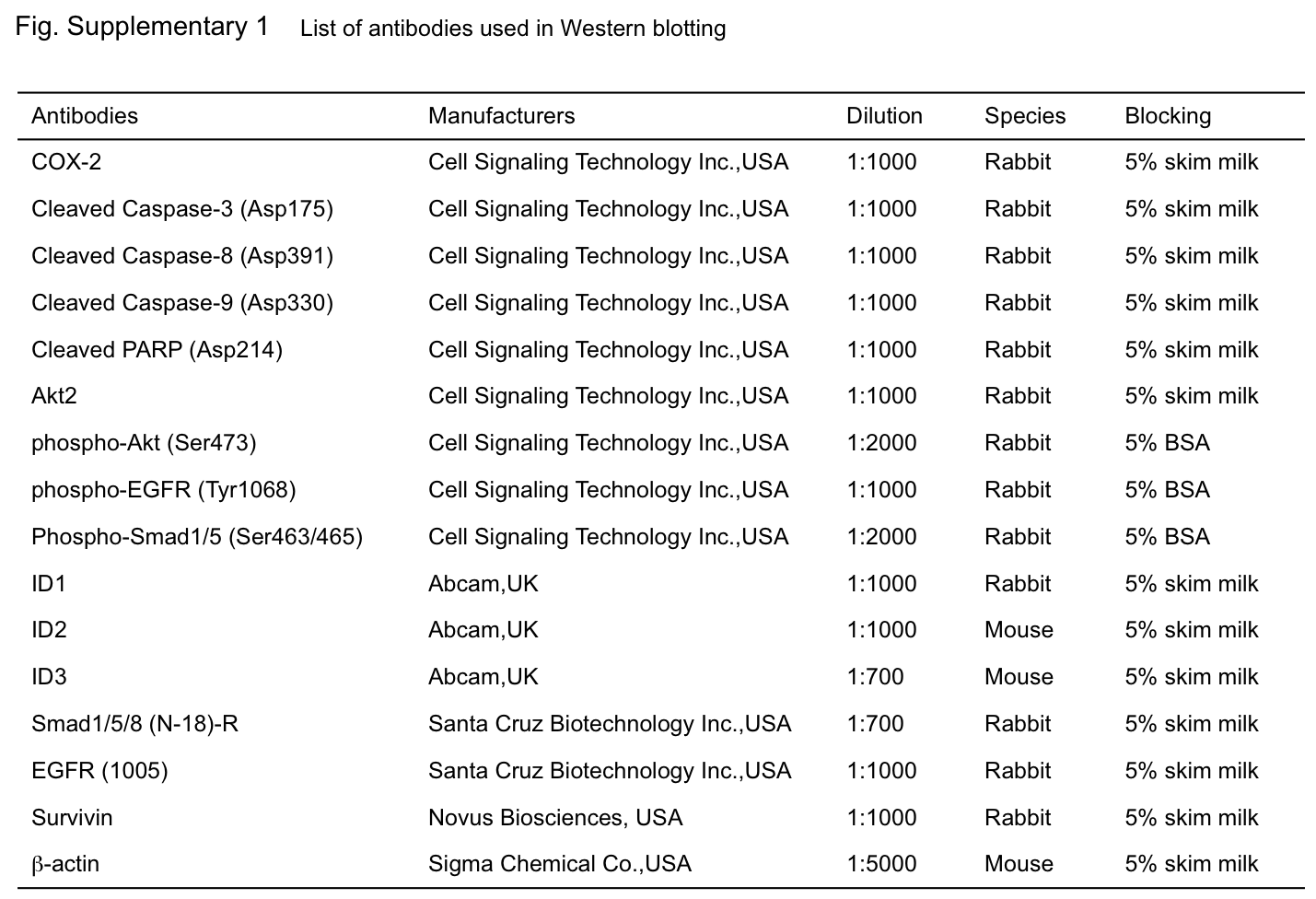


Fig. Supplementary 1 List of antibodies use Western blotting

Supplement: Supplementary file 1 — Supplementary material 1 (DOCX 225 KB) [file 11060_2017_2380_MOESM1_ESM.docx]
